# Tropisetron modulates peripheral and central serotonin/insulin levels *via* insulin and nuclear factor kappa B/receptor for advanced glycation end products signalling to regulate type-2 diabetes in rats

**DOI:** 10.1039/c7ra13105d

**Published:** 2018-03-27

**Authors:** Reem Ali Mohamed, Omneya Galal, Ahmed Refaat Mohammed, Hanan Salah El-Abhar

**Affiliations:** Department of Pharmacology and Toxicology, Faculty of Pharmacy, October University for Modern Sciences and Arts (MSA) 26 July Mehwar Road Intersection with Wahat Road 6th October City Cairo 12566 Egypt ralia@msa.eun.eg +20-002-01224611087; Department of Pharmacology and Toxicology, Faculty of Pharmacy, Cairo University Cairo Egypt

## Abstract

Despite its known central effect, 5% of serotonin is found centrally, while around 95% is found peripherally. Serotonin is stored and co-released with insulin upon pancreatic islets stimulation by glucose. This fact raises the curiosity regarding its possible role in diabetes. Hence, in this study, we assessed the possible modulatory effects of tropisetron, a 5-HT3 receptor antagonist, on type 2 diabetes mellitus models in rats. The rats were allocated into two groups: normal and diabetic. The latter group was treated with metformin (500 mg kg^−1^, p.o.), tropisetron (1 and 2 mg kg^−1^, i.p.), and a combination of metformin and tropisetron (1 mg kg^−1^). The different treatment regimens corrected glucose and lipid homeostasis manifested by the decrease in serum levels of glucose, fructosamine, homeostasis model of insulin resistance, triglycerides, total cholesterol, free fatty acid, as well as receptor for advanced glycation end products. Additionally, the treatments elevated levels of insulin, serotonin, and homeostasis model of β-cell function. On the molecular level, treatments corrected the altered insulin signaling cascade (phosphorylated insulin receptor substrate 1, phosphorylated protein kinase B, and glucose transporter 4), and inhibited β-catenin and phosphorylated nuclear factor kappa B p65 in the assessed soleus skeletal muscle. A similar pattern was duplicated in the hippocampus. This study provided evidence for the role of tropisetron on type 2 diabetes mellitus *via* modulating the insulin signaling cascade (insulin, phosphorylated insulin receptor substrate 1, phosphorylated protein kinase B, and glucose transporter 4), improving lipid/glucose profile, decreasing inflammatory markers (receptor for advanced glycation end products, and phosphorylated nuclear factor kappa B p65), as well as increasing 5-HT and reducing β-catenin.

## Abstract 2

Schematic illustration of tropisetron's (5-HT3 antagonist) modulation of the insulin and Wnt/β-catenin signaling pathways both peripherally and centrally in type 2 diabetes.

## Graphical abstract

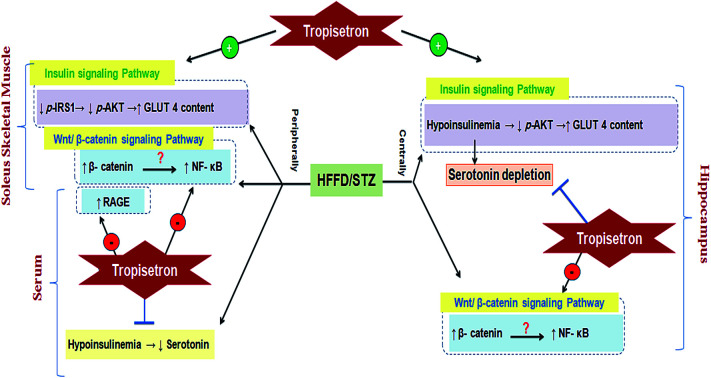

## 1. Introduction

Type 2 diabetes mellitus (T2DM) is the most common form of DM characterized by hyperglycemia, insulin resistance (IR), and relative insulin deficiency.^1^ Its insidious onset and late recognition are associated with increased morbidity and mortality.^2^ Most patients with T2DM are obese and obesity itself causes some degree of IR. Others who are not obese may have an increased percentage of body fat distributed mainly in the abdominal area.^3^

The primary hypothesized mechanisms to explain IR and islet β-cell dysfunction in T2DM involve increased oxidative stress, lipotoxicity, glucotoxicity and inflammatory responses.^4^ These stresses can result from sedentary life style and westernized food over nutrition.^5,6^ Skeletal muscle is one of the organs greatly affected by diabetes since it is strongly dependent on oxidative phosphorylation for energy production. In type 2 diabetes and obesity insulin resistance of skeletal muscle results in dysregulation of the oxidation of both carbohydrate and lipid.^7^

Apart from its known peripheral effects, diabetic patients experience cognitive dysfunction,^8^ depression,^9^ and anxiety^10^ associated with changes in brain structure that appear to develop over time, such as hippocampal injury, reduction in gray matter density, changes in white matter microstructure, and atrophy.^8^ This association between T2DM and brain function has usually been evaluated in elderly individuals having diabetes for many years, who generally have poor glycaemic control and significant vascular diseases. Interestingly, it was found that even patients with relatively well-controlled diabetes of less than 10 years from the time of diagnosis also had memory defects. These patients had significantly smaller hippocampal volumes compared to the control group and the memory defects were restricted to the hippocampal-based memory and not observed in the other cognitive domains.^11^ Hence, both the skeletal muscle and the hippocampus were chosen as targeted organs.

Serotonin is one of the central neurotransmitters that is largely found in peripheral tissues (∼95%); it is mainly synthesized in β-cells and is co-secreted along with insulin upon stimulation with glucose.^12^ However, its role in diabetes is a confusing issue due to the controversial findings. A previous study^13^ showed that both insulin and 5-HT decreased in a T2DM mouse model, while El-Marasy *et al.* (2014) reported that the antidepressant drug hesperidin used by diabetic depressive patients abated hyperglycemia and inflammation and elevated 5-HT level.^14^ On the contrary, other researchers reported that pregnant women who used selective 5-HT reuptake inhibitors (SSRI) gave birth to off springs with abnormal glycemic control^15^ and that 5-HT increases with inflammatory diseases including T2DM.^16^

The correlation between 5-HT and insulin is controversial since some studies reported a positive impact of 5-HT on insulin, while others did not. In 2009, Paulmann *et al.* reported that in normal conditions, 5-HT controls the insulin release by increasing the expression of tryptophan hydroxylase 1 mRNA in β-cells.^12^ Additionally, Amireault *et al.* (2012) stated that 5-HT induces the proliferation of β-cells and insulin by stimulating the 5-HT_1A_ receptor present on the pancreas, in addition to a receptor independent mechanism known as serotonylation.^17^ Moreover, in mammals, the onset of pregnancy is accompanied by an increase in insulin producing β-cells to provide sufficient insulin to support the growth of the fetus and the energy homeostasis of the mother.^18^ The tryptophan hydroxylase 1 mRNA, which is responsible for the synthesis of peripheral 5-HT, increases by almost 20 folds at this critical stage of pancreatic development.^19^ This expansion is mediated by several 5-HT receptors such as 5-HT_2B_ and 5-HT_1D._^20^ Moreover, 5-HT *per se* was reported to reduce the glucose-stimulated liberation of insulin in a concentration-dependent fashion.^21^ The use of tropisetron abolished the negative effect of 5-HT on insulin secretion.

One of the 5-HT receptor subtypes is the 5-HT3 receptor, which is unique among all monoamine receptors as it is a cation-selective chloride-channel that belongs to the ligand-gated ion-channel superfamily.^22^ This receptor is found peripherally^23^ and in the central nervous system with strong hybridization signals seen in the hippocampus.^24^ Evidence from both human and animal studies suggested that the 5-HT3 receptor antagonists, which were first developed as expeditious agents to counteract chemotherapy-induced emesis, possess a variety of neuroprotective and anti-inflammatory properties as documented in models of colitis,^25^ asthma,^26^ and rheumatic diseases.^27^

Although tropisetron, a 5-HT3 receptor antagonist, enhanced insulin release from insulin producing β-cell line,^21^ the exact mode of action regarding its secretagogue action remains unclear. An earlier study reported that tropisetron reduces the inward cation currents, particularly calcium cation, which is the main target for sulfonylureas, which are the classical insulin secreting agents.^28^ Having secretagogue action and anti-inflammatory characters, tropisetron was the drug investigated in the current study to unveil possible mechanisms in T2DM both centrally and peripherally using a T2DM rat model.

## 2. Materials and methods

### 2.1. Drugs and chemicals

Streptozotocin (STZ) was purchased from Sigma-Aldrich Co. (St. Louis, MO, USA) and tropisetron hydrochloride (Navoban®) was procured from Novartis pharmaceuticals (El Amiria, Cairo, Egypt). Metformin was a gift from CID Co. (Giza, Egypt) and long acting insulin (Insulatard®) was obtained from Novo Nordisk Co. (Copenhagen, Denmark). Cholesterol was procured from Panreac Co. (Barcelona, Spain), fructose (Unifructose®) was obtained from UNIPHARMA (Cairo, Egypt), citric acid and sodium citrate used for the preparation of citrate buffer were obtained from Al-Gomhoria Pharmaceutical Co. (Cairo, Egypt) and sheep fat was obtained from a commercial source. All other chemicals used were of analytical grade.

### 2.2. Animals

Adult male Wistar rats (90–120 g) were purchased from Animal Production Research Institute (Dokki, Giza, Egypt). They were housed in standard polypropylene cages and kept under constant environmental conditions of 12/12 h dark/light cycles at a temperature of 25 ± 2 °C. These conditions were kept constant until the end of the experiment. The rats were fed commercially available normal pellet diet and water *ad libitum* and were left for 5 days for acclimatization prior to the dietary manipulation. The rats were handled according to the recommendations in the Guide for the Care and Use of Laboratory Animals of the National Institutes of Health. The protocol was approved by the Committee of the Faculty of Pharmacy on the Ethics of Animal Experiments of the Cairo University (permit number: PT 1087). All efforts were made to minimize the suffering of the rats.

### 2.3. Development of insulin resistant/type 2 diabetic rats

The current model was a modification on a previously verified model described in a previous study.^29^ Briefly, 66 male Wistar rats were initially divided into two dietary regimens. Ten of them were separated in two cages (5 rats per cage) and fed a normal fat diet (NFD) [∼3000 kcal g^−1^: fat as oils (3%), protein (21%), carbohydrate as starch (60%), fibers (3%), vitamins and minerals (3%)] to serve as the normal control group, while the remaining 56 (4 rats per cage) were fed an in-house prepared high fat/high fructose diet (HFFD)) [∼5300 kcal g^−1^: fats as oils (3%) + (15%) sheep tail fat + (1%) cholesterol powder, protein (21%), carbohydrate as starch (60%), fibers (3%), vitamins and minerals (3%)] and permitted 20% fructose in drinking water. Dietary manipulation was continued for 12 weeks, after which the rats developed IR as indicated by elevated fasting blood glucose, triglycerides (TGs), total cholesterol (TC), and hyperinsulinemia. This state was further confirmed by intraperitoneal glucose tolerance test (IPGTT), homeostasis model assessment index (HOMA-IR), and AUC. Subsequently, only the HFFD rats (220 ± 40 g) with hyper-glycemia, -cholestrolemia, -triglyceridemia, and -insulinemia received daily single doses of long acting human insulin (Insulatard®) (0.5 IU kg^−1^, i.p) for 1 week to augment the state of IR and decrease the rate of mortality from STZ. At the beginning of week 14, NFD rats received a single dose of citrate buffer (i.p) and the HFFD group received a freshly prepared sub-diabetogenic dose of 35 mg kg^−1^ STZ dissolved in freshly prepared citrate buffer (pH 4.5). STZ was injected i.p. once after an overnight fasting to produce frank hyperglycemia and the rats also received 5% glucose in drinking water for the first 24 h after the STZ injection to guard against the initial STZ-induced hypoglycemia.^30^ After the STZ injection, all rats were fed NFD and fructose free water until the end of the study. A week after the STZ injection, the rats that had blood glucose levels between 11.1–19.43 mmol L^−1^, hyper-cholestrolemia, hyper-triglyceridemia, and hypo-insulinemia were considered as late stage insulin resistant/type 2 diabetic rats and were included in the study.^31^ During the development of this model, a weekly estimation of the body weight (BW) was recorded in addition to the levels of fasting serum glucose, TGs, TC, and insulin.

### 2.4. Intraperitoneal glucose tolerance test (IPGTT)

To confirm the state of insulin resistance, which is essential before the injection of STZ, the IPGTT (2 g glucose kg^−1^) was performed randomly on 6 rats from each group after 6 h of fasting.^32^ Blood droplets were withdrawn from the tip of the tail vein at 0 time (before glucose) and every 30 min for 2 h after glucose administration. Blood glucose level was assessed using test strips for blood glucose testing device (Bionime®). IR was reflected by the IPGTT and was further confirmed by the fasting hyper-insulinemia and the HOMA-IR value.

### 2.5. Experimental design

The investigator was not blind to the experimental groups to determine the effects of the different treatment regimens. Out of 56 rats on HFFD, 14 died (∼25%) and 2 rats were resistant (∼4.8%). Rats on NFD were set as the 1^st^ group (*n* = 10) and received only the vehicles. Diabetic rats were randomly divided into five groups (*n* = 8). The 2^nd^ group served as the insulin-resistant/T2D control, while the 3^rd^, 4^th^ and 5^th^ groups received metformin at 500 mg kg^−1^, p.o.,^33^ and tropisetron hydrochloride at two dose levels 1 mg kg^−1^ (ref. 34) (Trop_1_) and 2 mg kg^−1^ (ref. 25) (Trop_2_) i.p, respectively. The rats in the 6^th^ group received a combination of Trop_1_ and Met. All the treatments were sustained for 15 days and the last dose of any treatment was given 24 h before the rats were euthanized. Rats were fasted for 18 h before the time of death to minimize the feeding-related variations in the lipid and glucose patterns.
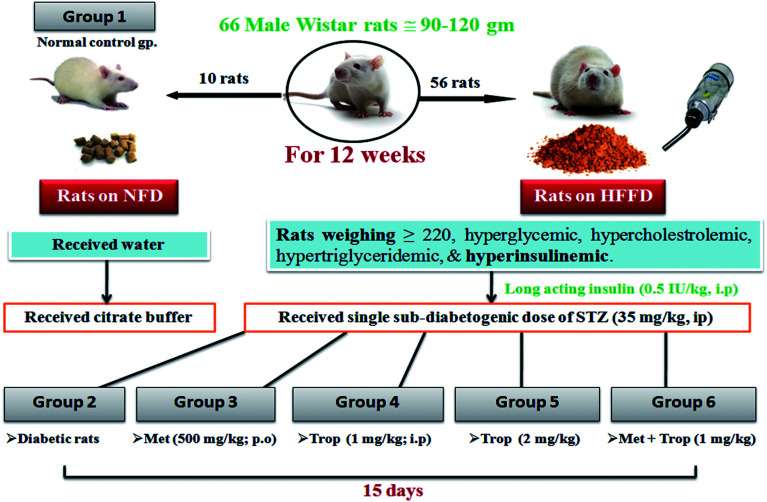


### 2.6. Collection of blood samples

At the time of carnage, blood was collected from the jugular veins of thiopental anaesthetized rats and was centrifuged (800 × *g*, 4 °C, 20 min) to separate the sera. ELISA technique was carried out using the corresponding ELISA kit (MYBioSource, CA, USA) for the assessment of fructosamine (Cat# MBS704663), insulin (Cat# MBS045315), serotonin (Cat# MBS166089), the receptor for advanced glycation end-products (RAGE; Cat# MBS495003) and free fatty acids (FFAs; Cat# MBS704474). Glucose level in addition to serum lipid profile, *viz.*, TGs and TC were measured calorimetrically using SPINREACT kit (Girona, Spain). Finally, HOMA-IR and HOMA-β, which were used to assess β-cell function, were calculated according to the following equations, respectively.^35^
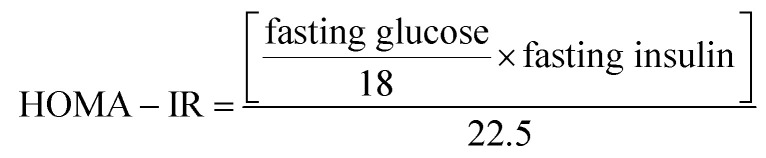

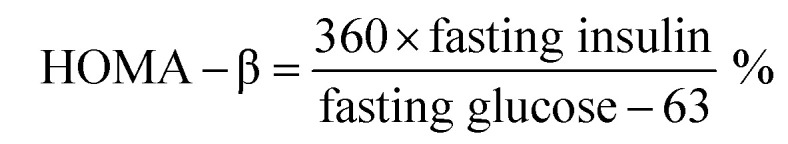


### 2.7. Tissue extracts

Following blood collection, the rats were euthanized by administering a thiopental overdose. Both hippocampi and soleus skeletal muscle were harvested, washed, dried, weighed, homogenized in phosphate buffer saline (10%) and kept in aliquots for the estimation of the following biomarkers using the corresponding rat ELISA kits (MYBioSource, CA, USA). Contents of glucose transporter 4 (GLUT 4; Cat#MBS2023267), phosphorylated Akt (p-Akt; Cat# MBS700138), phosphorylated (Ser 536)-nuclear factor kappa-B p65 (p-NF-κB p65, Cat# MBS722507), and β-catenin (Cat# MBS727964) were assessed in the muscle and hippocampus. In addition, the amount of phosphorylated insulin receptor substrate 1 (pIRS1, Cat# MBS919486) was measured in the muscle and the hippocampal contents of 5-HT (Cat# MBS166089) and insulin (Cat# MBS045315) were determined in the brain besides measuring their levels in blood. All parameters were assessed according to the manufacturer's protocol.

### 2.8. Statistical analysis

Values are expressed as mean ± S.E. of 6 rats and the differences between the groups were tested for significance using analysis of variance (ANOVA), followed by Tukey's Multiple Comparison test as the *post hoc* test. The GraphPad prism software, version 6 (GraphPad Software Inc., San Diego, CA, USA) was used to analyze the data and to draw the attached figures. The level of statistical significance was accepted at *P* < 0.05.

## 3. Results

### 3.1. Development of T2D model

**Fig. 1:**
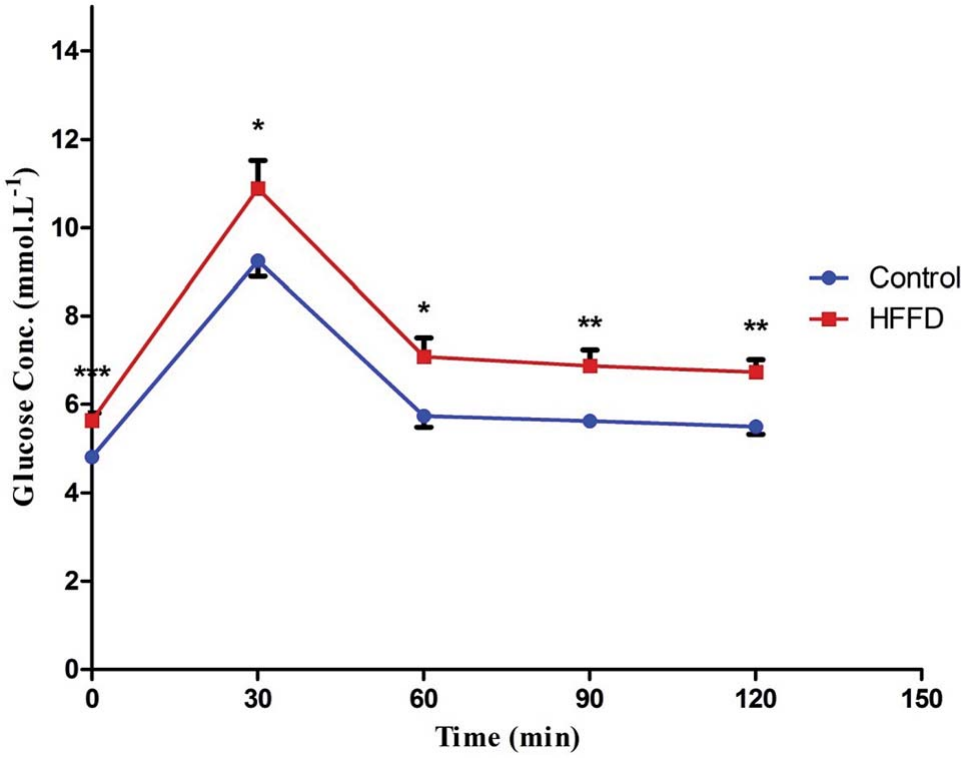
Effect of 13 weeks of high fat fructose diet (HFFD) intake on IPGTT in rats. Values are expressed as mean ± S.E of 6 rats as compared to normal control group (*) (unpaired *t*-test), *P* < 0.05. Line graph showing the effect of high fat fructose diet + insulin (HFFD/insulin) on glucose tolerance test. The rats on HFFD/insulin exhibited an upward shift in the curve along the 2 h period, indicating a state of insulin resistance.

Table 1 shows that HFFD alone developed a state of IR, which was manifested by a significant increase in glucose, insulin, HOMA-IR, AUC and HOMA-β, which are effects that were confirmed by the IPGTT (Fig. 1). The IPGTT test showed that the HFFD rats presented the same pattern displayed by those with NFD during the 2 h period, but with a significant upward shift in the curve, indicating a state of glucose intolerance/IR. After the administration of STZ (Table 2) frank hyperglycemia was detected along with increased levels of TGs, TC and HOMA-IR. However, this was accompanied by hypo-insulinemia and a decrease in HOMA-β and BW as compared to the normal control group.

**Table 1:** The effect of high fat fructose diet (HFFD) on serum levels of glucose, insulin, HOMA-IR, AUC, and HOMA-β in rats^a^

Parameters/groups	Glucose (mmol L^−1^)	Insulin (μIU mL^−1^)	HOMA-IR	AUC	HOMA-β
Normal control	4.80 ± 0.04	5.170 ± 0.1215	1.080 ± 0.043	248.8 ± 5.64	36.88 ± 0.911
HFFD	5.63 ± 0.17*	13.32 ± 1.125*	3.335 ± 0.316*	298.0 ± 9.83*	72.74 ± 3.707*

a Values are expressed as mean ± S.E of (6 rats). Statistical analysis was performed using unpaired student's *t*-test; (*) indicates significant difference from normal control group at (*p* < 0.05). HOMA-IR: homeostasis model assessment of IR; HOMA-β: homeostasis model assessment of β-cell function; HFFD: high fat-fructose diet; NFD: normal fat diet.

**Table 2:** The effect of high fat high fructose diet/Streptozotocin (HFFD/STZ) on BW, glucose, insulin, triglycerides (TG), and total cholesterol (TC), HOMA-IR and HOMA-β in rats^a^

Groups/parameters	Normal control	1 week after STZ
Body weight (g)	299.8 ± 8.411	256.5 ± 10.67*
Glucose (mmol L^−1^)	4.18 ± 0.06	17.24 ± 1.23 *
Insulin (μIU ml^−1^)	43.89 ± 1.964	19.70 ± 1.612 *
TG (mmol L^−1^)	3.82 ± 0.08	4.98 ± 0.24 *
TC (mmol L^−1^)	4.18 ± 0.18	8.15 ± 0.62*
HOMA-IR	8.171 ± 0.3638	14.79 ± 1.589*
HOMA-β	405.0 ± 28.32	28.61 ± 5.225*

a Values are expressed as mean ± S.E of (6 rats). The rats were fed high fat diet (HFD) and were allowed 20% fructose in drinking water throughout a period of 13 weeks. At the start of week 14, diet manipulation was stopped and the rats were injected with STZ. After the STZ injection and until the end of the study, the rats received normal fat diet (NFD) and fructose free water. Statistical analysis was performed using unpaired student's *t*-test, where (*) significant difference from normal control group at (*p* < 0.05). BW: body weight; HOMA-IR: homeostasis model assessment of IR; HOMA-β: homeostasis model assessment of β-cell function.

### 3.2. Effect of different treatment regimens on parameters of glucose homeostasis and serotonin

**Fig. 2:**
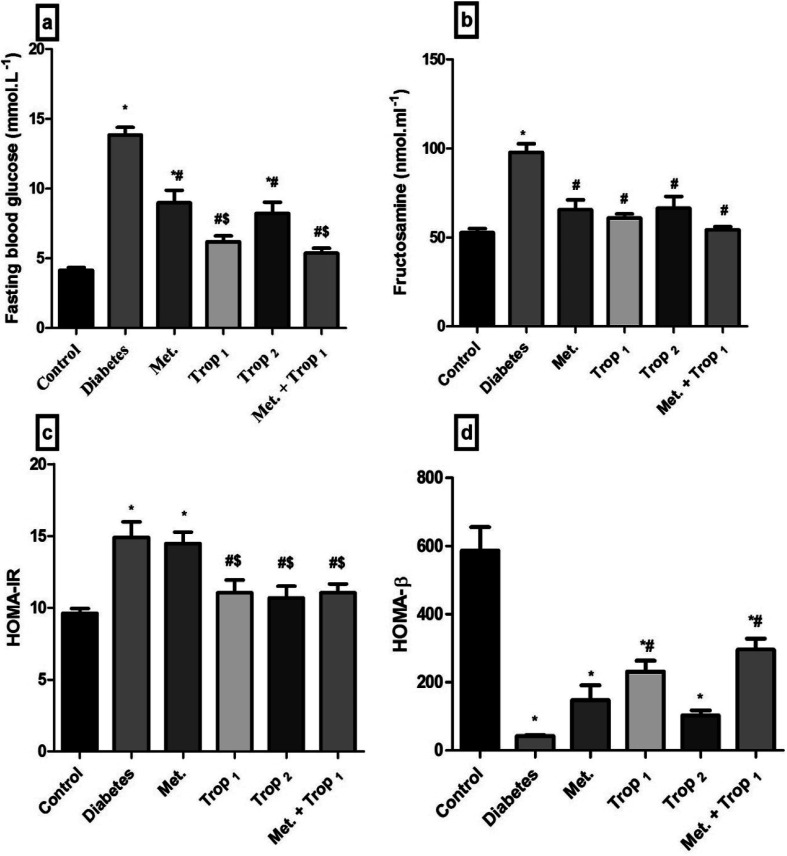
Effect of diabetes and 15 day administration of Met (500 mg kg^−1^), Trop_1_, Trop_2_ or Met + Trop_1_ on serum (a) glucose, (b) fructosamine, (c) HOMA-IR, and (d) HOMA-β in rats. Statistical analysis was performed using one-way ANOVA, followed by Tukey's *post hoc* test (*P* < 0.05). Values are expressed as mean ± S.E of (6 rats) and a comparison was obtained for normal control (*), diabetic control (#), and Met ($) treated groups. Met: metformin (500 mg kg^−1^, p.o.); Trop_1_: tropisetron (1 mg kg^−1^, i.p); Trop_2_: tropisetron (2 mg kg^−1^, i.p).

**Fig. 3:**
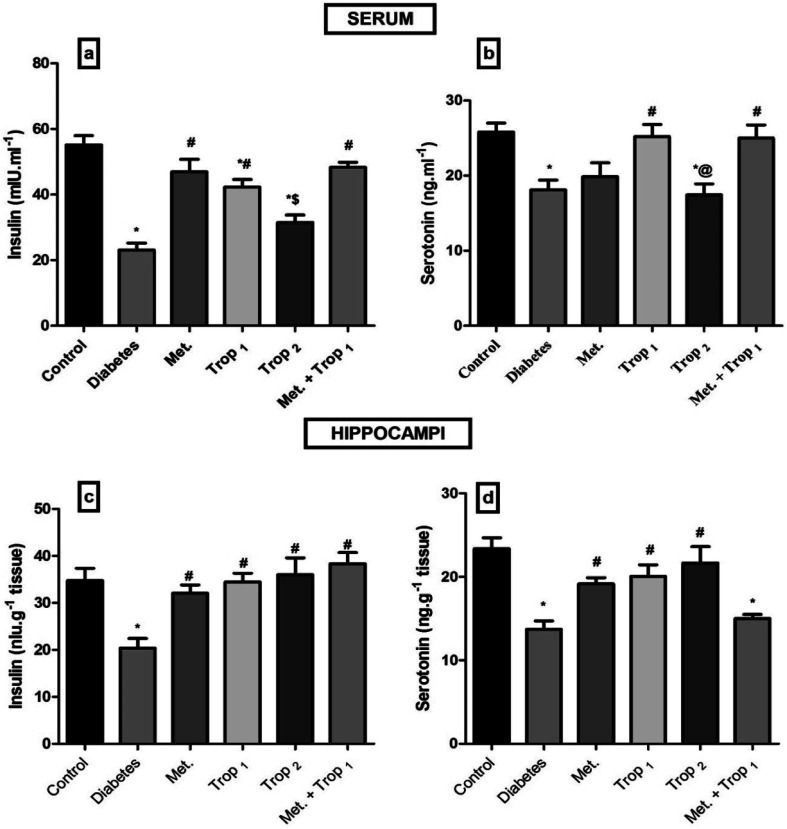
Effect of diabetes and 15 day administration of Met (500 mg kg^−1^), Trop_1_, Trop_2_ or Met + Trop_1_ on serum (a and b) and hippocampal (c and d) contents of insulin and 5-HT in rats. Statistical analysis was performed using one-way ANOVA, followed by Tukey's *post hoc* test (*P* < 0.05). Values are expressed as mean ± S.E of (6 rats) and a comparison was obtained for normal control (*), diabetic control (#), Met ($) and Trop_1_ treated (@) groups. Met: metformin (500 mg kg^−1^, p.o.); Trop_1_: tropisetron (1 mg kg^−1^, i.p); Trop_2_: tropisetron (2 mg kg^−1^, i.p).

As depicted in Fig. 2, Trop_1&2_ reverted the high serum levels of (A) glucose, (B) fructosamine, and (C) HOMA-IR; however, (D) HOMA-β was corrected only by Trop_1_ and the combination regimen. Fig. 3 illustrates that T2D model abated both serum and hippocampal levels of insulin and 5-HT. Trop_1_ and its combination elevated both serum (A) insulin and (B) 5-HT, while Met increased the levels of only serum insulin. In the hippocampus, although all treatments increased (C) insulin and (D) 5-HT contents, 5-HT content was not affected by the combination regimen.

### 3.3. Effect of different treatment regimens on body weight and parameters of lipid profile

**Fig. 4:**
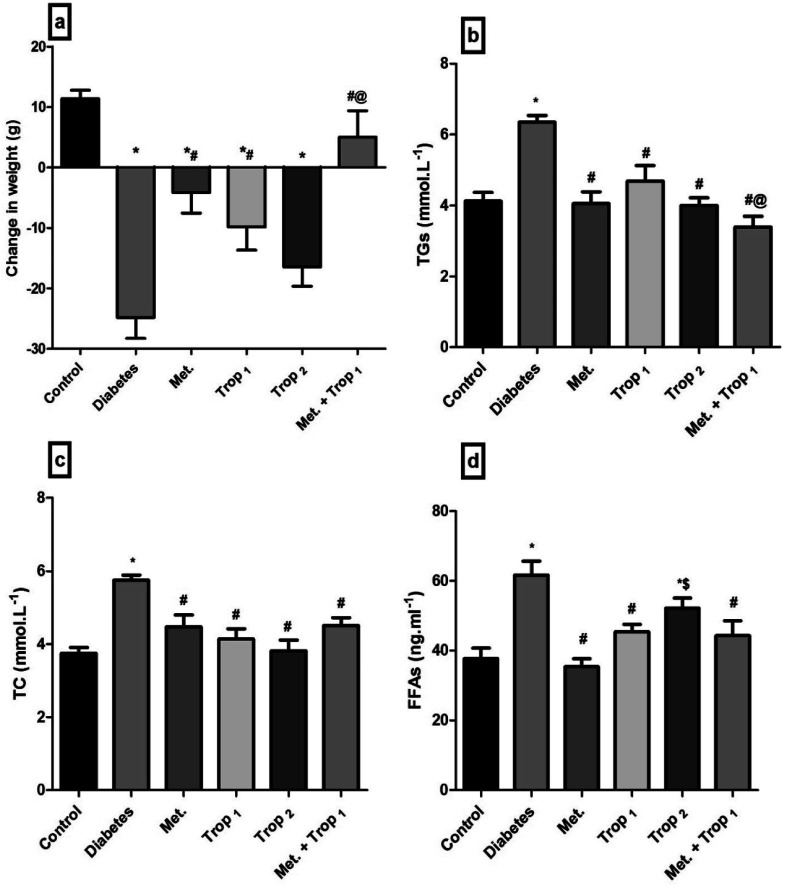
Effect of diabetes and 15 day administration of Met (500 mg kg^−1^), Trop_1_, Trop_2_ or Met + Trop_1_ on BW (a) serum TG (b), TC (c) and (d) FFAs in rats. Statistical analysis was performed using one-way ANOVA, followed by Tukey's *post hoc* test (*P* < 0.05). Values are expressed as mean ± S.E of (6 rats) and a comparison was obtained for normal control (*), diabetic control (#), Met ($) and Trop_1_ treated (@) groups. Met: metformin (500 mg kg^−1^, p.o.); Trop_1_: tropisetron (1 mg kg^−1^, i.p); Trop_2_: tropisetron (2 mg kg^−1^, i.p).

Diabetic rats experienced a significant weight loss (24.83 g) as compared to the control group rats which gained weight during the two-week treatment period. Administration of different treatment regimens hindered diabetes-induced weight loss to different extents to reach a normal level in the combination regimen treated group (Fig. 4a). Moreover, dyslipidemia, characterized by (B) hypertriglyceridemia, (C) hypercholesterolemia, and (D) hyper-FFAs, was detected in diabetic rats; nevertheless, almost all treatments normalized these biomarkers.

### 3.4. Effect of different treatment regimens on inflammatory parameters

**Fig. 5:**
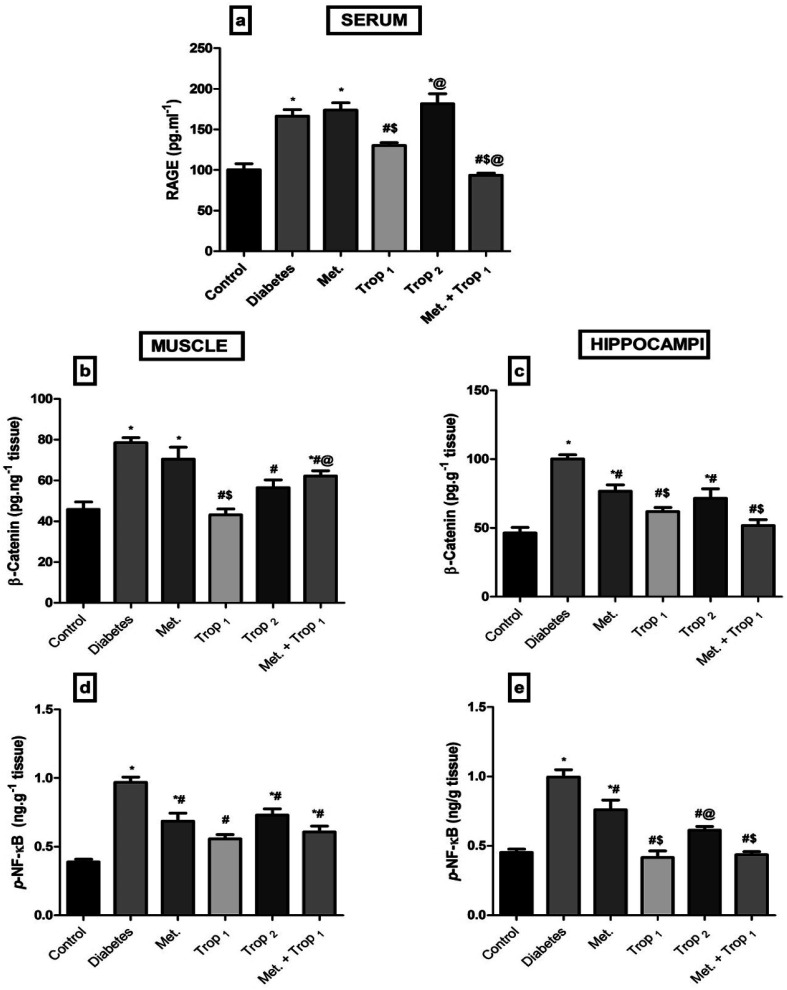
Effect of diabetes and 15 day administration of Met (500 mg kg^−1^), Trop_1_, Trop_2_ or Met + Trop_1_ on serum (a) RAGE, muscular and hippocampal (b and c) β-catenin, muscular and hippocampal (d and e) contents of p-NF-κB in rats. Statistical analysis was performed using one-way ANOVA, followed by Tukey's *post hoc* test (*P* < 0.05). Values are expressed as mean ± S.E of (6 rats) and a comparison was obtained for normal control (*), diabetic control (#), Met ($) and Trop_1_ treated (@) groups. Met: metformin (500 mg kg^−1^, p.o.); Trop_1_: tropisetron (1 mg kg^−1^, i.p); Trop_2_: tropisetron (2 mg kg^−1^, i.p).

The current model also elevated (Fig. 5a) serum RAGE (65.87%) as well as (B) muscular and (C) hippocampal contents of β-catenin by 71.26% and 116.27%, respectively. This effect extended to increased muscular (D) and hippocampal (E) contents of p-NF-κB p65 (149.4% and 120.4%, respectively). These altered parameters were mainly corrected by Trop_1_ and its combination.

### 3.5. Effect of different treatment regimens on insulin signaling

**Fig. 6:**
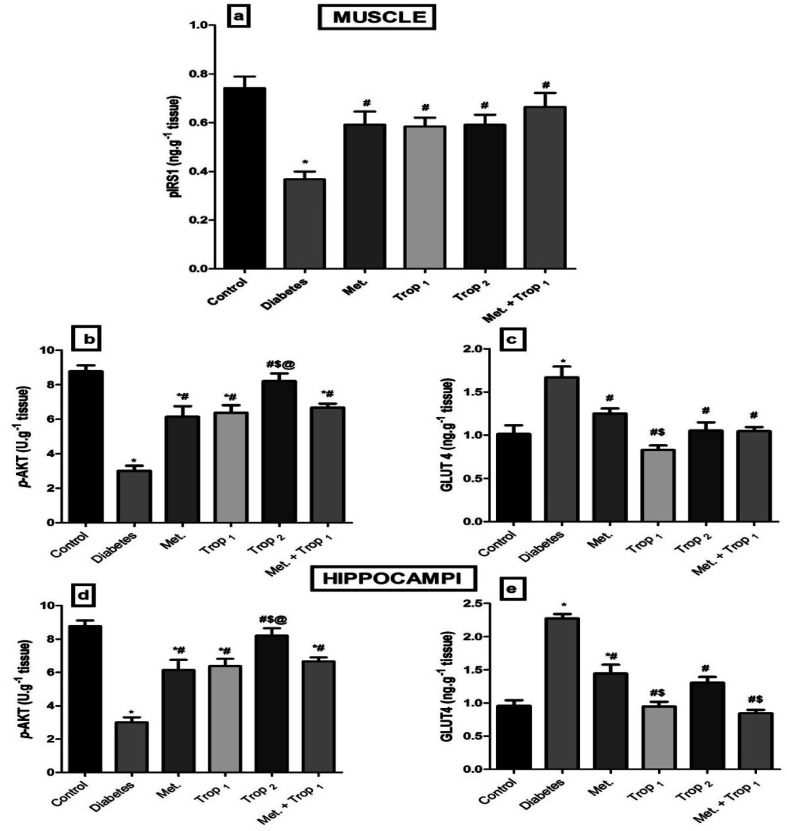
Effect of diabetes and 15 day administration of Met (500 mg kg^−1^), Trop_1_, Trop_2_ or Met + Trop_1_ on muscular content of (a) pIRS1, (b) p-AKT, (c) GLUT4, and hippocampal content of (d) p-AKT, and (e) GLUT4 in rats. Statistical analysis was performed using one-way ANOVA, followed by Tukey's *post hoc* test (*P* < 0.05). Values are expressed as mean ± S.E of (6 rats) and a comparison was obtained for normal control (*), diabetic control (#), Met ($) and Trop_1_ treated (@) groups. Met: metformin (500 mg kg^−1^, p.o.); Trop_1_: tropisetron (1 mg kg^−1^, i.p); Trop_2_: tropisetron (2 mg kg^−1^, i.p).

The current model interrupted the insulin signaling pathway (Fig. 6) as evidenced by the sharp decline in the (A) muscular pIRS1 and (B) p-Akt with an increase in the cellular content of (C) GLUT4. This pathway was affected similarly in the hippocampus, where diabetes caused a notable decline in the hippocampal content of (D) p-Akt and an increase in (E) GLUT4. The different treatments improved the muscular contents of pIRS1 and p-Akt, while they lowered GLUT4 significantly. However, in the hippocampus, all treatments except Trop_2_ increased p-Akt, while all treatments abated GLUT4 content with Trop_1_ and the combination, indicating a more pronounced action.

## 4. Discussion

T2DM is a complex disease associated with multi-pathological states, such as hyper-triglyceridemia, impaired glucose tolerance, IR^36^ and inflammation.^4^ Insulin is known to be co-localized with 5-HT in the same granules along with the fact that they are co-released when pancreatic islets are stimulated. This sheds light on the role played by 5-HT and its receptors in diabetes.^37^

The development of an animal model that can imitate the natural sequence of events that occur in diabetic patients is essential for the reliability of the study and the results generated through it. This sequence started with the transition from a healthy to a pre-diabetic state characterized by either impaired fasting glucose or impaired glucose tolerance or both^38^ due to the presence of dyslipidemia and IR.^39^ This pre-diabetic state was emulated by the administration of HFFD for 13 weeks, after which the rats were dyslipidemic with IR and consequent hyperinsulinemia.

The second transition was from pre-diabetic to frank T2DM; this progression required the loss of a significant portion of functional β-cell mass.^40^ This would eventually lead to hypo-insulinemia and persistent hyperglycemia.^41^ In the current model, the transition to frank T2DM was achieved using a sub-diabetogenic dose of STZ (35 mg kg^−1^), which caused a relative but significant destruction of β-cells (*P* < 0.0001) that was accompanied by persistent hyperglycemia (>200 mg dl^−1^) along with hypo-insulinemia.

The present study is the first to highlight the anti-diabetic effect of Trop, the 5-HT3 receptor antagonist. Particularly in the low dose, Trop reverted the diabetes-associated alterations, abated weight loss, and leveled off glucose, fructosamine, and HOMA-IR. Additionally, it increased HOMA-β and insulin level. Trop also improved the lipid profile manifested by a decrease in serum TGs, TC and FFAs, as well as the inflammatory parameters RAGE and p-NF-κB. These effects were partially mediated through insulin and Wnt/β-catenin signaling pathways.

The anti-diabetic effect of Trop, proved herein, is in line with a previous *in vitro* study, in which Trop enhanced insulin release from insulin producing β-cell line.^21^ Moreover, ondansetron, another 5-HT3 receptor antagonist, was previously reported to revert hyperglycemia induced by the central activation of serotonergic system, indicating the involvement of 5-HT3 receptor subtype in this hyperglycemic effect.^42^ Trop also normalized both fructosamine and RAGE levels. These effects can be linked to the restoration of the glucose level, during which fructosamine is formed as a result of glucose-induced protein modification and the advanced glycation end products (AGE) are formed as a result of fructosamine oxidative cleavage.^43^ Hence, a decrease in AGE can be partially responsible for the decrease of its receptor RAGE as documented herein and previously.^44^

Insulin level, which is a critical indicator of IR, was reduced in both serum and hippocampal tissue of the diabetic control rats, resembling a late stage of T2DM.^31,41,45^ In this study, the use of Trop_1_ restored serum insulin level, while both doses elevated the cerebral insulin content. This improvement could be attributed to the hypoglycemic effects of Trop since hyperglycemia *per se* induces β-cell death and impairs insulin secretion. Similarly, the improvement in the lipid profile, which will be discussed later, plays a role, during which T2DM-induced elevation in FFAs impairs β-cell secretory function, induces β-cell death, and IR.^46^ Hence, Trop-mediated insulin restoration can be partially explained by combating gluco-lipotoxicity. A recent study by Gupta *et al.* (2016) supports our findings and they have graphically summarized the influence of 5-HT3 antagonist on glucose/insulin homeostasis.^9^

In the present study, synchronization between the two hormones insulin and 5-HT was detected, during which the levels of both decreased in serum and hippocampus of the diabetic rats. However, contradictory findings were reported regarding the 5-HT levels in diabetes. In line with our results, several studies have reported a decline in cerebral levels of 5-HT in animal models of diabetes^47^ and in the blood of diabetic patients^48^ as well as in STZ-diabetic rats.^49^ The persistent hyperglycemia along with insulin deficiency causes depletion of 5-HT and subsequently the serotonergic function in the brain. Previous researches^50,51^ referred this depletion to the decreased content of its precursor tryptophan (TRP), which was supported by a study reporting a decrease in serum TRP of diabetic patients.^52^ These facts directed several authors to suggest that changes in free plasma level of TRP influence that in the brain^53–55^ and these alterations may be behind the diabetes-associated memory impairment.^52^ The decrease in TRP level in brain and the consequent decrease in 5-HT level may be attributed to the diabetes-mediated elevation of large neutral amino acids in the plasma^50,56^ that compete with TRP for entry into the brain.^57^ Another clarification was offered by Russo *et al.*,^58^ who referred the decrease in brain 5-HT level to the greater metabolism of TRP by alternative pathways, such as liver TRP-oxygenase enzyme activity, which increases in diabetic condition.^58,59^ Alternatively, other authors revealed contradictory results; they reported an elevation in plasma serotonin level in T2DM patients.^48^

Treatment with Trop elevated serum level of 5-HT and its cerebral content. Recently, another 5-HT3 receptor antagonist also reversed the diabetes-induced reduction in 5-HT levels in different brain regions.^9^ This effect was abolished upon using a 5-HT3 agonist, thus proving the involvement of 5-HT3 blockage in the elevation of 5-HT.

Wnt signaling pathway has a definite role in metabolic homeostasis and has been studied in several diabetic models.^60,61^ In the present study, a high glucose level was accompanied by an elevation in β-catenin, pointing to the activation of the Wnt pathway; this result is in line with those stated in previous reports.^62,63^ However, whether the activation of this pathway is a protective response or an injurious response is still debatable.

Authors who support the protective role showed that the Wnt pathway was activated to promote the regeneration of damaged pancreatic islet cells^62^ or healing of diabetic wounds.^64^ In contrast, other authors reported the harmful effects of the activated β-catenin, such as in the induction of peritoneal injury, followed by fibrosis^65^ and the occurrence of diabetic nephropathy-induced renal interstitial fibrosis.^61^

In the current study, muscular/hippocampal β-catenin activation was accompanied by an increase in the inflammatory transcriptional factor p-NF-κB, possibly backing the harmful effect; the same co-elevation was documented in other inflammatory conditions.^66–68^ Hence, Trop revealed its anti-inflammatory effect *via* inhibiting p-NF-κB and RAGE, possibly through the inactivation of the Wnt pathway documented herein as validated by the decreased content of β-catenin.

The anti-diabetic effect of Trop can also be attributed partly to the improved insulin signaling pathway. Trop enhanced the muscular content of pIRS1 and consequently p-Akt. The latter is responsible for the translocation of GLUT4 to allow the uptake of glucose from blood. Hence, the diabetes-induced increase in GLUT4 content in both organs might be a sort of compensatory mechanism, where although the content was increased, it apparently failed to reach cell membrane with the reduced content of p-Akt; earlier reports support the present finding.^69,70^

This study was the first to investigate the effect of Met on hippocampal content of 5-HT. Some of the gastrointestinal side effects observed during treatment with Met were thought to be produced by the release of 5-HT and other neurotransmitter substances within the duodenal mucosa.^71^ These authors found that the binding of the released serotonin to 5-HT3 receptors was responsible for the gastrointestinal side effects experienced with Met treatment. It is known that the administration of 5-HT3 receptor agonists induces vomiting and diarrhea in experimental animals.^72^ Similarly, the stimulation of 5-HT3 receptors located on vagal visceral afferents by 5-HT, released from gut enterochromaffin cells, is associated with gastrointestinal side effects.^73,74^ In a similar scenario, the present study reported a 5-HT elevation in Met treated group, which attained a significant level only in the hippocampus, which may explain the side effects experienced with the general use of Met. Therefore, the administration of 5-HT3 antagonists including Trop could prevent these symptoms and are used as anti-emetics by virtue of the blockage of 5-HT3 receptor. Hence, the addition of Trop to Met could abolish these symptoms by decreasing the effects of serotonin on 5-HT3 receptor. Although the combination regimen failed to elevate cerebral 5-HT level, it did not entail the insulin level or alter the other anti-diabetic effects of the combination regimen.

Met is a standard anti-diabetic drug. Therefore, almost all our findings are in accordance with previously reported studies on glucose and lipid panels,^75^ RAGE,^76^ insulin signaling pathway, and p-NF-κB,^77^ as well as serum content of β-catenin.^78^ Regarding the effect of Met on the insulin level, previous studies in addition to the current study have shown that Met could indirectly increase insulin secretion by combating the toxic effects of high glucose and FFAs on β-cells, which will eventually lead to improved secretory function.^46^ In a previous pancreatic islets *in vitro* study, the authors highlighted the improved insulin secretion in Met treated islets. They concluded that Met is able to restore the intracellular abnormalities of glucose and FFA metabolism and could restore a normal secretory pattern in rat pancreatic islets, whose secretory function has been impaired by chronic exposure to elevated FFA or glucose levels.^79^

Compared to Met alone, the combination regimen significantly inhibited serum glucose and RAGE levels as well as the levels of hippocampal GLUT4, β-catenin, and p-NF-κB. In addition, the combination regimen had a prominent effect on BW, where it completely diminished the weight loss and the rats even started to gain weight. However, the other parameters tended to be improved, but did not reach a significant level.

## 5. Conclusion

In conclusion, the present study showed that Trop and the modulation of 5-HT3 receptors have a positive role in treatment of T2DM. This was achieved, at least in part, through the amelioration of inflammation and the modulation of two signaling pathways, *viz.*, insulin and Wnt signaling cascades, which improved insulin sensitivity both peripherally and centrally. However, future investigations are required to unveil other potential mechanisms involved in the anti-diabetic action of Trop.

## Conflicts of interest

There are no conflicts of interest to declare.

## Supplementary Material
